# The Association Between Adverse Childhood Experiences and Postpartum Depression

**DOI:** 10.3389/fgwh.2022.898765

**Published:** 2022-05-26

**Authors:** Danielle M. Prentice, Banan W. Otaibi, Christy Stetter, Allen R. Kunselman, Serdar H. Ural

**Affiliations:** ^1^Department of Obstetrics and Gynecology, Division of Maternal Fetal Medicine, Penn State Health Milton S. Hershey Medical Center, Hershey, PA, United States; ^2^Penn State College of Medicine, Hershey, PA, United States; ^3^Department of Public Health Sciences, Division of Biostatistics and Bioinformatics, Penn State College of Medicine, Hershey, PA, United States

**Keywords:** maternal mental health, antepartum mood disorders, adverse childhood experiences, obstetrics, pregnancy, postpartum

## Abstract

**Objective:**

Adverse childhood experiences (ACEs) are linked to worsening overall health outcomes and psychological diagnoses. Routine screening, particularly in patients with postpartum depression (PPD), would identify patients who could benefit from interventions to prevent the perpetuation of ACEs and establish a system of preventative care to mitigate the risks of adverse health outcomes associated with high ACE scores. The purpose of this study is to explore the link between ACEs and PPD to advocate for the use of the ACE questionnaire as a routine screening tool in all pregnant patients diagnosed with PPD. We hypothesize that a cohort of patients with PPD will be more likely to have high-risk ACE scores than the general female population.

**Study Design:**

Our IRB approved, retrospective cohort study identified all patients diagnosed with PPD at an academic medical center between January 2015 and December 2019. The subjects were identified using retrospective chart review. Subjects were recruited via telephone and asked to complete an ACE questionnaire. Questionnaires were sent via RedCap. ACE scores were calculated, categorized as 0, 1, 2, 3, or 4 or more ACEs, and compared to the prevalence in the original Kaiser-CDC ACE study female cohort using a chi-square goodness-of-fit test.

**Results:**

There were 132/251 surveys completed (53% response rate). In our PPD population, 19.3% had 0 ACEs, 17.0% had 1 ACE, 13.1% had 2 ACEs, 16.5% had 3 ACEs, and 34.1% had 4 or more ACEs. These percentages were significantly different from the Kaiser-CDC ACE Study percentages of 34.5, 24.5, 15.5, 10.3, and 15.2%, respectively (*p* < 0.001).

**Conclusion:**

Our unique study showed that women with PPD are more likely to have high-risk ACE scores than the general female population. This finding has important implications in regards to counseling, intervening to prevent perpetual ACEs, and establishing important provider-patient relationships for life-long preventative care.

Non-gendered language is used when possible throughout. However, the wording from studies cited in this paper was preserved.

## Introduction

The importance of the postpartum period or the fourth trimester of pregnancy has only recently become a significant focus of maternal health. Included in the leading causes of maternal mortality in the United States are drug overdose and suicide, most of which occur in the postpartum period. Maternal mental health plays a significant role in both (1). Postpartum depression (PPD), more recently termed perinatal depression, is a serious mood disorder affecting one in eight pregnant patients. This varies by region, with some states reporting a prevalence of one in five. Additionally, the prevalence of PPD has increased by 0.22% annually between 2012 and 2018 (2). As reported by the Center for Disease Control, one in five pregnant patients were not asked about depression symptoms during their pregnancy and one in eight were not asked about symptoms during the postpartum period (2). The strongest identifiable risk factors for PPD include antenatal or prenatal depression, low self-esteem, high stress levels, lack of social support, and previous history of depression or other mood disorders (3). While knowledge of the exact nature of the relationship between trauma exposure and perinatal depression is limited, research suggests that exposure to traumatic events increases the risk for PPD (3–5). Research conducted by Zhang and colleagues demonstrates various types of violence including sexual, emotional, physical, domestic, and childhood violence all significantly increase the risk of developing PPD (6).

Adverse childhood experiences (ACEs) were first described in relation to health outcomes by a large study conducted by Kaiser-Permanente and the CDC. Felitti and colleagues were the first to evaluate the impact of adverse childhood experiences on health outcomes in adulthood. They defined adverse childhood experiences (ACEs) as sources of stress that individuals experience early in life, usually before age 18, including abuse (physical, sexual, and psychological), neglect, and family dysfunction (7). ACEs have since been linked to psychological disorders, substance abuse, risky sexual behavior, obesity, cardiovascular disease, cancer, and diabetes (7–10). Recent research has investigated the links between adverse childhood experiences and changes in biomarkers including inflammation (e.g., CRP), cardio/metabolic systems (e.g., BMI), genetics (e.g., telomere length), and endocrine systems (e.g., cortisol) that may explain their long-term health consequences (11). These increased risks are seen with each additional ACE, however, the most dramatic increase in risk is seen at “high-risk” ACE scores deemed to be 4 or more (10). ACEs have also been linked to premature all-cause mortality (12). It is also important to note that there is an intergenerational perpetuation of ACEs that is seen among families. This means that the children of parents with high-risk ACE scores are more likely to have high-risk ACEs themselves. This makes intervention to prevent the perpetuation of ACEs all the more important (13).

Recognizing the impact of adverse childhood experiences on overall health, recent research has assessed the impact of childhood adversity on maternal health in the perinatal and postpartum periods. Women with higher ACE scores are more likely to have unplanned pregnancies (14). Furthermore, they are at a greater risk of developing various complications of pregnancy including gestational diabetes, preeclampsia, and preterm labor and delivery (15). Women with higher ACEs are also more likely to experience psychosocial difficulties during pregnancy, increasing the risk of medical and neurodevelopmental problems in their infants (16, 17). Additionally, attention has begun to turn to the impact ACEs may have on maternal mental health. Several studies show that pregnant people with ACEs, especially higher ACE scores, are at an increased risk of developing PPD (18, 19). Research shows that patients with higher ACE scores may have depressive symptoms longer despite traditional treatment (20).Routine prenatal ACE screening may be a valid tool to identify pregnancy health risks, but more research is needed to determine the most effective methods of screening and intervention for pregnant patients with high scores (21).

The aim of this study was to compare the prevalence of ACEs in our PPD population to the prevalence of ACEs in the female population in the original Kaiser-CDC study in order to evaluate the potential role of perinatal ACE screening as an important capture point for ACE-based therapy, intervention to prevent the mitigation of ACEs, and establishing long-term preventative care.

## Materials and Methods

This study is a retrospective cohort study that is IRB approved (STUDY00014270) by the Penn State Health Milton. S Hershey Internal Review Board. Verbal consent was obtained over the telephone which was approved by the IRB.

All patients diagnosed with PPD between January 2015 and 2019 were identified using the electronic medical record. ICD-10 codes were used to create the patient database. The code utilized was F53.0 (postpartum depression) The Edinburgh Postnatal Depression Scale was used for PPD screening and diagnosis was made by the postpartum provider. At our institution, postpartum providers include certified nurse midwives, physician assistants, obstetricians, family medicine providers, and perinatologists. Chart reviews were conducted to determine whether inclusion or exclusion criteria were met. Inclusion criteria were study participant of 18 years or older, follow up in the postpartum period at one of our institution's clinic locations, a diagnosis of PPD, and English speaking. Exclusion criteria were pregnancy outcome of neonatal death or stillbirth, history of mental health disorder or psychiatric diagnosis prior to pregnancy, and non-English speaking subjects.

Those who met criteria for participation were contacted via telephone. The overall aim of the study was discussed with the patient in detail. They were then asked if they would like to complete the validated ACE questionnaire (Supplementary Table 1). The ACE questionnaire used was validated in the Kaiser-CDC ACE study and the questions included were chosen because they were found to be strongly linked to negative outcomes (7). Informed consent was obtained verbally. Once consent was obtained, the patient and their electronic mail address were entered into the REDCap (v.9.5.33) application. Chart review was performed for demographic information and entered into REDCap. Questionnaires were then sent via the REDCap (v.9.5.33) application. If a subject failed to respond within 72 h, a reminder was sent. No further telephone contact was made.

ACE scores were calculated from our data, categorized as 0, 1, 2, 3, or 4, or more ACEs, and compared to the prevalence in the original Kaiser-CDC ACE study female cohort using a chi-square goodness-of-fit test. A cut-off of 4 or more was used to do the fact that this score is considered “high-risk” and is found to be associated with the most negative outcomes (8). To address survey nonresponse bias, the analysis used propensity score weighting (22). Five auxiliary variables were used to determine the weighting: age, ethnicity, race, mode of delivery, and parity. Analyses were conducted using SAS version 9.4 (SAS Institute Inc., Cary, NC).

## Results

Overall, 251 subjects were eligible, of which 132 participated in this study. Respondents had a mean age of 32 ± 5.7 years (range 21–48 years) and were primarily Caucasian (87.9%). Patient characteristics for respondents vs. non-respondents are shown in Table 1 in which differences of statistical significance (*p* < 0.05) were seen between age and race.

**Table 1:** Demographic characteristic of respondents vs. non-respondents.

	**Respondents (n = 132)**	**Non-Respondents (*n* = 119)**	***P*-value**
Age (years)	32.5 ± 5.7	30.1 ± 5.7	0.001
Hispanic ethnicity	5 (3.8%)	11 (9.2%)	0.08
Race			
*White*	116 (87.9%)	89 (74.8%)	0.01
*Non-white*	10 (7.6%)	13 (10.9%)	
*Unknown*	6 (4.6%)	17 (14.3%)	
Vaginal delivery	94 (71.2%)	81 (68.1%)	0.59
First birth	84 (63.6%)	63 (52.9%)	0.09

*Data are reported as mean ± SD with two-sample t-test or n (%) with chi-square test*.

**Figure 1:**
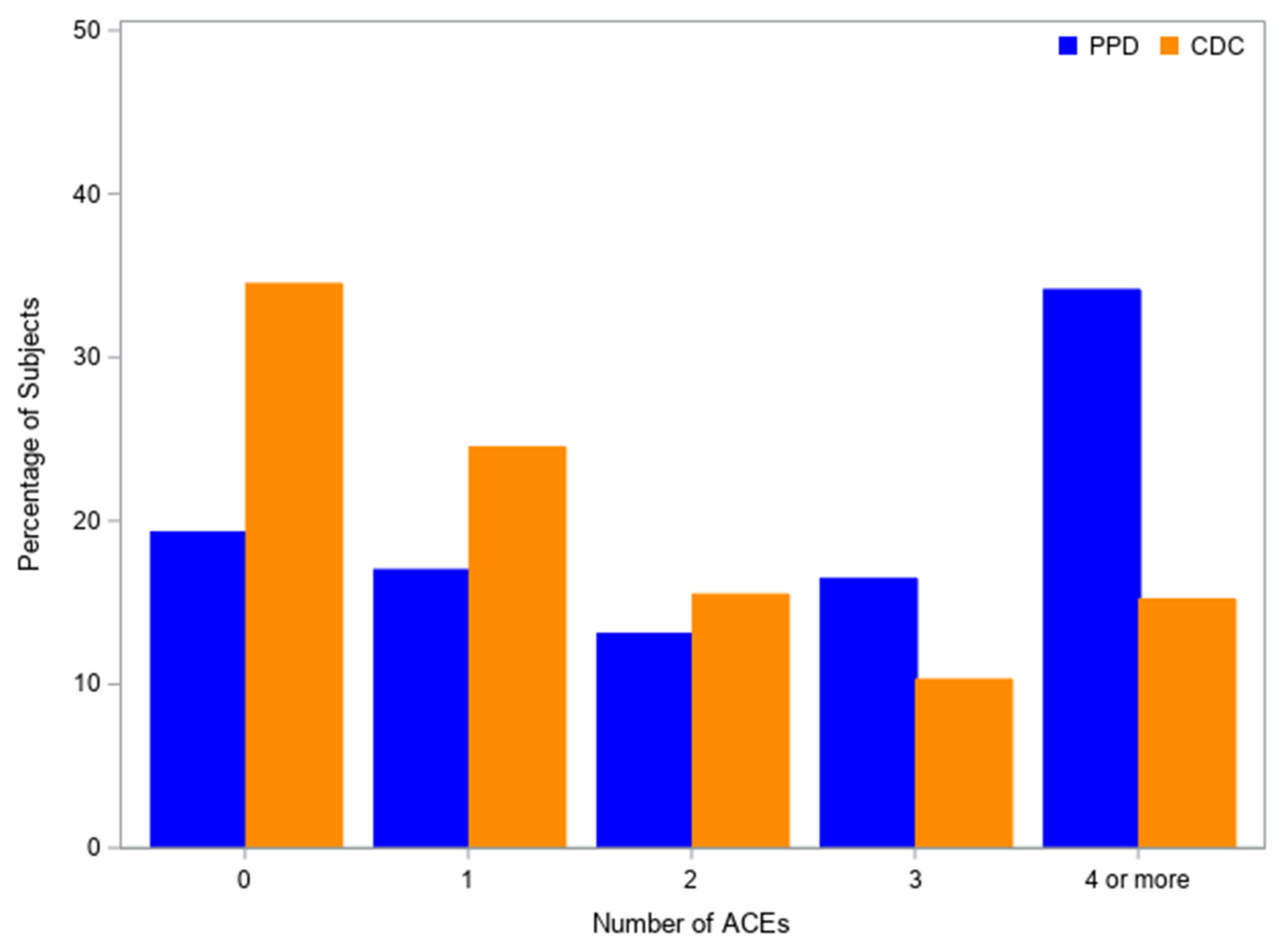
Kaiser-ACE study female population vs. postpartum depression population.

In our PPD population, 26 respondents had 0 ACEs, 24 had one ACE, 18 had 2 ACEs, 22 had 3 ACEs, and 42 had four or more ACEs. After weighting to account for survey nonresponse, the percentages for these ACE categories were 19.3, 17.0, 13.1, 16.5, and 34.1%. ACE score prevalence for the female cohort of the CDC-Kaiser ACE Study (*n* = 9,367) was 34.5, 24.5,15.5, 10.3, and 15.2%, respectively (7). These percentages were significantly different from our PPD cohort (*p* < 0.001) (Figure 1). The 4 or more ACE category was the primary contributor to the chi-square statistic, accounting for 64% of the statistic. The 0 ACE category contributed 18% to the chi-square statistic. Therefore, we can infer that our PPD population has a higher percentage of participants in the four or more ACE category and lower percentage of participants in the 0 ACE category compared to the Kaiser-CDC female population.

Figure 1 shows the comparison between the adverse childhood experience (ACE) scores in the female cohort of the Kaiser-CDC Adverse Childhood Experience study and our postpartum depression population. PPD is based on weighted percentages.

## Discussion

The postpartum population studied has a significantly higher prevalence of high-risk ACE scores than the original Kaiser-CDC ACE study. This is an important finding given the relationship between ACEs and healthcare disparities. This research is timely and critical as maternal mortality rates, particularly maternal suicide rates, continue to climb. In fact, mental health conditions including suicide, overdose, and unintentional injuries are a leading cause of death in all pregnant patients (23). While ACE screening has not been widely adopted and applied to all pregnant patients or all patients diagnosed with postpartum depression, our findings add to literature advocating for its use in clinical practice. Researchers in Calgary similarly demonstrated ACEs to be predictive of depressive symptoms in pregnancy, both across the perinatal period and during the postpartum period (24). Additionally, several studies have linked ACEs to postpartum depression in patients with low-socioeconomic status (19, 25). However, it is likely, based on the results of this study, that ACEs counts are higher among our pregnant patient, therefore screening should be widened to include the entirety of the population. Screening patients with PPD for ACEs may assist in improving overall individual and community health. Recent research highlights that ACEs impact both the person who experienced them and their children, highlighting an intergenerational perpetuation of ACEs and their long-term health consequences (13). This process occurs in multiple different pathways. Adults who experienced ACEs are more likely to engage in behaviors that are known to impair parenting and child health such as tobacco use, substance abuse, and risky sexual behaviors (7). Additionally, ACEs have been associated with mental health disorders which may also impair parenting (13), highlighting the utility of targeting antenatal mood disorders. Targeted therapy can be utilized to address childhood trauma and work to mitigate the generational effect of ACEs. For example, a study in British Columbia, utilizing a different validated questionnaire named the Antenatal Psychosocial Health Assessment, promoted targeted PPD screening in women with past or current experiences of interpersonal violence (26). Community programs can be developed in order to target the perpetuation of ACEs based on a particular population's data. Patients can be referred to the appropriate preventative care providers based on their high-risk ACE score associated health disparities, hopefully decreasing their increased risk of morbidity and early mortality.

The strengths of this study include a relatively high survey response rate of 53% with a total of 132 subjects participating in the study. A large number of patients expressed interest in this area of research and were grateful that their histories were being taken into account. This was not unique to our study; researchers in Wisconsin assessed the feasibility of implementing ACE screening into regular primary care practice by conducting a study of parent/guardian perception of discussing ACEs during regular visits (27). Their findings suggest conversation about ACEs is generally well received and could easily be integrated into regular conversation with patients. Our experience was similar in that patients were apt to discuss their experiences.

The limitations of this study include its retrospective nature and survey-based design, which introduces the possibility of nonresponse bias. To account for survey nonresponse bias we weighted the responding cases to better reflect the target population, but this may not sufficiently account for differences in ACE scores between respondents and non-respondents. Additionally, our study population was very homogenous with 87.9% of survey respondents being Caucasian. Non-responders were more likely to be from minority populations, although due to the homogenous makeup of our population, this was small sample size. This is an important limitation for a few reasons. One reason is that minority populations have an increased risk of poor obstetrical outcomes and exploration of the impact of ACEs on this patient population in the postpartum period is important. Secondly the homogeneity of our population may limit the reproducibility to more heterogeneous populations.

Future research directions in this area are abundant. We are currently planning a prospective cross-sectional study in which all patients are recruited at the beginning of their pregnancy and asked to complete an ACE questionnaire. Patients will be followed through 6 weeks postpartum with the aim of comparing ACE scores in patient who develop peripartum mood disorders to those who do not. Furthermore, expanding to centers with a more heterogenous population would aid in determining which demographic risk factors may impact the findings of our study. Additionally, this topic is ripe for qualitative research. Patients' experiences with ACEs and peripartum mood disorders are important, and feedback relating to their overall needs at the time is instrumental in developing effective interventions and community programs.

## Conclusion

Patients with postpartum depression have higher ACE scores than the general female population. ACE based interventions should be developed to improve overall individual health, mitigate healthcare disparities, and reduce the generational perpetuation of ACEs.

## Data Availability Statement

The raw data supporting the conclusions of this article will be made available by the authors, without undue reservation.

## Ethics Statement

The studies involving human participants were reviewed and approved by Penn State Health Milton S. Hershey Internal Review Board. Written informed consent for participation was not required for this study in accordance with the National Legislation and the Institutional requirements.

## Author Contributions

DP, BO, and SU contributed to conception and design of the study. BO organized the database. CS and AK performed the statistical analysis. DP wrote the first draft of the manuscript. CS wrote sections of the manuscript. All authors contributed to the article and approved the submitted version.

## Conflict of Interest

The authors declare that the research was conducted in the absence of any commercial or financial relationships that could be construed as a potential conflict of interest.

## Publisher's Note

All claims expressed in this article are solely those of the authors and do not necessarily represent those of their affiliated organizations, or those of the publisher, the editors and the reviewers. Any product that may be evaluated in this article, or claim that may be made by its manufacturer, is not guaranteed or endorsed by the publisher.

## Supplementary Material

Supplementary Table 1 Adverse Childhood Experience Survey. Questionnaire obtained from (7).
