# Supplementary material for: The Association Between Adverse Childhood Experiences and Postpartum Depression
Source: Front Glob Womens Health. 2022 May 26;3:898765. doi: 10.3389/fgwh.2022.898765 (PMC9183059; doi:10.3389/fgwh.2022.898765)
Supplement: Supplementary Table 1 — Adverse Childhood Experience Survey. Questionnaire obtained from (7). [file Table_1.DOCX]

| Supplemental Table 1: Adverse Childhood Experience Survey |
| --- |
| 1. Did a parent or other adult in the household often or very often… Swear at you, insult you, put you down, or humiliate you? or Act in a way that made you afraid that you might be physically hurt? |
| 1. Did a parent or other adult in the household often or very often… Push, grab, slap, or throw something at you? or Ever hit you so hard that you had marks or were injured? |
| 1. Did an adult or person at least 5 years older than you ever… Touch or fondle you or have you touch their body in a sexual way? or Attempt or actually have oral, anal, or vaginal intercourse with you? |
| 1. Did you often or very often feel that … No one in your family loved you or thought you were important or special? or Your family didn’t look out for each other, feel close to each other, or support each other? |
| 1. Did you often or very often feel that … You didn’t have enough to eat, had to wear dirty clothes, and had no one to protect you? or Your parents were too drunk or high to take care of you or take you to the doctor if you needed it? |
| 1. Were your parents ever separated or divorced? |
| 1. Was your mother or stepmother: Often or very often pushed, grabbed, slapped, or had something thrown at her? or Sometimes, often, or very often kicked, bitten, hit with a fist, or hit with something hard? or Ever repeatedly hit over at least a few minutes or threatened with a gun or knife? |
| 1. Did you live with anyone who was a problem drinker or alcoholic, or who used street drugs? |
| 1. Was a household member depressed or mentally ill, or did a household member attempt suicide? |
| 1. Did a household member go to prison? |
